# The Growing Challenge of Chronic Kidney Disease: An Overview of Current Knowledge

**DOI:** 10.1155/2023/9609266

**Published:** 2023-03-01

**Authors:** Rikke Borg, Nicholas Carlson, Jens Søndergaard, Frederik Persson

**Affiliations:** ^1^Department of Nephrology, Zealand University Hospital Roskilde, Roskilde, Denmark; ^2^Institute of Clinical Medicine, University of Copenhagen, Copenhagen, Denmark; ^3^Department of Nephrology, Rigshospitalet, Copenhagen, Denmark; ^4^Department of Research, The Danish Heart Foundation, Copenhagen, Denmark; ^5^Research Unit for General Practice, Department of Public Health, University of Southern Denmark, Odense, Denmark; ^6^Steno Diabetes Center Copenhagen, Herlev, Denmark

## Abstract

Chronic kidney disease (CKD) is becoming one of the world's most prevalent noncommunicable chronic diseases. The World Health Organization projects CKD to become the 5th most common chronic disease in 2040. Causes of CKD are multifactorial and diverse, but early-stage symptoms are often few and silent. Progression rates are highly variable, but patients encounter both an increased risk for end-stage kidney disease (ESKD) as well as increased cardiovascular risk. End-stage kidney disease incidence is generally low, but every single case carries a significant burden of illness and healthcare costs, making prevention by early intervention both desirable and worthwhile. This review focuses on the prevalence, diagnosis, and causes of CKD. In addition, we discuss the developments in the general treatment of CKD, with particular attention to what can be initiated in general practice. With the addition of recent landmark findings and the expansion of the indication for using sodium–glucose cotransporter 2 inhibitors, there are now new effective treatments to add to standard therapy. This will also be relevant for primary care physicians as many patients with CKD have their family physician as their primary health care professional handling kidney function preservation. In the future, more precise and less invasive diagnostic methods may not only improve the determination of the underlying cause of CKD but may also carry information regarding which treatment to use (i.e. personalized medicine). This could lead to a reduced number of preventive treatments per individual, while at the same time improving the prognosis. This review summarizes ongoing efforts in this area.

## 1. Introduction

With this paper, we aim to provide an overview of chronic kidney disease with a focus on recent developments in treatment possibilities and the need for collaboration across healthcare sectors to improve detection, treatment, and prognosis.

The prevalence of chronic kidney disease (CKD) is increasing globally, with CKD projected to become the fifth most prevalent chronic condition by 2040 [1]. Global incidence and prevalence of CKD vary depending on divergent definitions of disease, different health care systems, social distributions, and risk factors for CKD, with the current standardized prevalence of CKD (eGFR < 60 ml/min/1.73 m^2^) estimated to be 10–15% [2–4].

The impact of CKD is multifold. Progression towards end-stage kidney disease (ESKD) (the final stage of kidney failure, requiring either chronic dialysis or kidney transplantation) entails a concurrent substantial increase in the risk of cardiovascular disease. An analysis of the Kaiser Permanente Renal Registry (*n* = 1,120,295) [5] found a marked increase in the age-standardized risk of death, cardiovascular events, and hospitalizations in individuals with an eGFR below 45 ml/min/1.73 m^2^. The risk of death from any cause was 0.76 per 100 person-years in individuals with eGFR above 60 ml/min/1.73 m^2^ but was 4.76 per 100 person-years in individuals with eGFR between 30 and 44 ml/min/1.73 m^2^. In parallel, the risk of cardiovascular events was 2.11 per 100 person-years in individuals with eGFR above 60 ml/min/1.73 m^2^ but was 11.29 per 100 person-years in individuals with eGFR between 30 and 44 ml/min/1.73 m^2^. This contributes to comorbidity and the burden of disease for many individuals with CKD, leading to an increase in both the number of medications and hospital visits. In a Scottish primary care study [6], it was demonstrated that 98.2% of adults with CKD had at least one comorbidity, versus 51.8% in controls. Hypertension, heart failure, diabetes, and coronary heart disease were found to be the most frequent concordant conditions. Furthermore, despite substantial improvement in both our understanding of the pathophysiology but also the treatment of cardiovascular risk and disease throughout the past decades, genuine advances in patients with advanced CKD including end-stage renal disease have remained elusive [7]. Plausibly, patients with advanced CKD have been systematically excluded from participation in large prospective clinical trials, leading to a lack of documentation of the benefit of treatment due to a paucity of evidence with implications direct for patient care [6, 8, 9].

Adding to the impact, the cost of renal replacement therapy (RRT), both dialysis and transplantation constitutes some of the most significant expenses in hospital-based health care. Based on annualized estimates of cost ascertained from national health care registers in Sweden, kidney disease is associated with a substantial economic burden, with costs elevated 4-fold, >10-fold, and >30-fold in patients with CKD, transplanted patients and patients on chronic dialysis, respectively [10]. The economic burden is predominantly driven by expenses related to excess hospitalization and dialysis treatment, each contributing >20% and >50% of total healthcare spending, respectively; with overall cost accounting for 1-2% of total healthcare spending in the UK [10, 11]. The mean prevalence of end-stage renal disease treatment is currently 750 per million population globally, with the cost of treatment—and annual attributable cost of treatment—closely associated with the national income level per capita [12]. Notably, the prevalence of treated end-stage renal disease has increased globally in spite of stagnant incidence rates in developed countries; plausibly due to increasing incidences in developing countries, overall improvement in survival rates, demographic population shift, and growth in prevalence of risk factors for end-stage renal disease including diabetes [13].

Survival in end-stage kidney disease is poor, annual mortality is estimated to be >10%, with five-year survival at approximately 50%. Cardiovascular disease continues to be the largest sole contributor to excess mortality in patients with end-stage kidney disease, with the attributable risk of cardiovascular death estimated to be >20-fold greater compared with general populations. Arrhythmias and sudden cardiac death continue to account for >30% of mortality in end-stage kidney disease, with an incidence estimated to be >25-fold greater compared with general populations. Due to the general exclusion of patients with advanced CKD from cardiovascular trials, evidence supporting current therapies remains uncertain. The implications are clear, and although a steady increase in treatment probability amongst patients with severe renal insufficiency has been noted, adherence to standard therapies in patients with CKD and cardiovascular disease including reperfusion therapy in myocardial infarction remains less implemented [14–17].

As expected, and well-known to many clinicians, this chronic and often silent condition affects the quality of life, not only among patients but also among their caretakers. In a large French survey [18], health-related quality of life in 2,693 patients with CKD stage 3–5 was compared with responses from 20,574 responders representing the general population. Health status was perceived as fair or poor by 27% of patients with CKD3, 44% of those with CKD4-5, and 43% of those receiving dialysis. Corresponding results were 12% of transplant recipients and 3% in the general population. Importantly, in a systematic review by Gilbertson et al. [19] comprising an international collection of studies, it was demonstrated that caregivers and partners of individuals on dialysis experience significant disease burden and reduced quality of life, further adding to the impact of the condition.

## 2. Diagnosis

The initial diagnosis of CKD is simple as it is based on biochemical parameters, i.e., an estimated glomerular filtration rate using a measure of plasma creatinine, with the help of an equation such as the CKD-EPI [20], which takes age, gender, and race into consideration. Recently, an update to the equation has been published [21], looking into more detail on the race-dependent differences, with a suggestion to include cystatin c in future measurements. There is however at present no international consensus on the wide endorsement of the latest version of the equation. Specific definitions of CKD can vary but seems to have only minor effects on global prevalence. An investigation of six different laboratory-based classifications of CKD used in a register study found differences in CKD incidence and prevalence between the different classifications used, but it did not impact mortality and ESKD rates [22].

CKD can be caused by many different diseases. Some are defined by distinct kidney diseases with renal pathology features (eg. glomerulopathies) while in other cases may be more of a result or a secondary complication to other acute or chronic conditions (eg. diabetes or hypertension). To help distinguish between different causes of CKD, urine analysis is important. Measurement of albuminuria/proteinuria provides essential information on the type of CKD, on prognosis, and can also be used as a target for treatment. Additional diagnostic tools are kidney ultrasound, specific blood samples, and in selected or severe cases a kidney biopsy to provide the true pathology for CKD.

For CKD in diabetes, there is some variation as to the diagnosis used. Diabetic kidney disease (DKD) is defined by elevated albuminuria, with a urinary albumin excretion rate (UACR) >300 mg/g in at least two out of three consecutive samples. The presence of retinopathy and the absence of other known kidney diseases supports the diagnosis. In addition, there is also a growing focus on persons with diabetes with nonalbuminuric impaired kidney function, perhaps with a more mixed etiology, and with less risk of progression. This is usually termed kidney disease in diabetes [23].

## 3. Treatment

Treatment for CKD can be divided into specific and general approaches (Table 1). Specific targeted treatments are available for only a fraction of patients with CKD depending on the accurate diagnosis. This is relevant e.g. for patients with glomerulonephritis where specific treatments with glucocorticoids or antibody therapies are recommended.

For the majority of patients with CKD, pharmacological treatment entails a more general approach nondependent on the pathology of CKD. The general form of treatment is rarely curative, but instead aims at slowing the progression of the disease and delaying kidney failure. Although many attempts have been made to develop new therapies for CKD, standard therapy supported by the best evidence remains blood pressure control and employment of blockers of the renin-angiotensin system (RAS), i.e., angiotensin-converting enzyme inhibitors (ACEi) or angiotensin receptor blockers (ARB). In addition to their blood pressure-lowering effects, these drugs are thought to have specific anti-inflammatory and antifibrotic effects of benefit in CKD. In various degrees of nephropathy in both types of diabetes, the collaborative group study [24] and the IRMA2 [25], IDNT [26], and the RENAAL study [27] demonstrated clear benefits with regards to delaying the progression of kidney outcome. In nondiabetic CKD the REIN [28] and the benazepril studies [29] demonstrated comparable results, backed up overall by findings in the more wide-ranging HOPE study [30].

Importantly, many of these studies also demonstrated cardiovascular protection in these high-risk populations, further securing RAS-blockade as standard therapy in guidelines.

In recent years, the emergence of sodium-glucose cotransporter 2 inhibitors (SGLT2i) has added results from a number of trials, with the majority being in type 2 diabetes, showing added benefit on top of standard care. The first dedicated kidney study was the CREDENCE study [31], testing canagliflozin 100 mg once daily in persons with diabetic nephropathy. The study was stopped prematurely after an interim analysis and showed a 30% relative reduction in the primary composite outcome of end-stage kidney disease (dialysis, transplantation, or a sustained eGFR of <15 ml/min/1.73 m^2^), a doubling of the serum creatinine, or death from renal or cardiovascular causes. In 2020, the DAPA-CKD study followed [32], with a mixed study population consisting of participants with diabetes and CKD as well as CKD without diabetes. The primary outcome was a composite of a sustained decline in the eGFR of at least 50%, end-stage kidney disease, or death from renal or cardiovascular causes. The study demonstrated a 39% relative reduction in the primary outcome, which was present in participants with diabetes, prediabetes, and nondiabetes [33] as well as in participants with diabetic nephropathy and nondiabetic CKD [34]. In fact, results in subgroup analyses of chronic glomerulonephritides (i.e. IgA nephropathy) were significant and offer novel therapeutic options in these conditions [34, 35].

SGLT2 inhibition is therefore included in recent guideline updates [36] as standard therapy in individuals with CKD with eGFR 25–75 ml/min/1.73 m^2^ and albuminuria, and dapagliflozin has expanded its label to include CKD. There are already indications from real-world data in type 2 diabetes that the use of SGLT2 inhibitors is associated with reduced eGFR decline and a lower number of kidney-related outcomes. The findings of the CVD-REAL 3 study [37], a multinational observational cohort with more than 65000 patients, suggested that results from the randomized controlled trials are transferable to a more general population. In a propensity-matched analysis, patients that initiated SGLT2i demonstrated a lower rate of eGFR decline and lower risk of major adverse kidney outcomes, as compared to people on other glucose-lowering drugs. Pending more widespread implementation of SGLT2i as kidney preventive treatment, these results should bode well also for the global CKD population.

Recently, the nonsteroidal mineralocorticoid receptor antagonist finerenone was added to guideline-recommended [38] therapy in persons with type 2 diabetes and CKD. This was done following two randomized controlled trials demonstrating benefits in relation to both cardiovascular and kidney outcomes [39, 40]. Ongoing trials on heart failure and nondiabetic CKD will perhaps broaden the use in the future.

It can be difficult to monitor the effect of initiated preventive treatment in CKD. To monitor eGFR requires serial measurements in stable conditions during 1-2 years for reliable assess the individual slope of kidney function decline. In proteinuric CKD however, a change in urinary albumin excretion (albuminuria) following initiation of treatment can be used as a prognostic indicator, as demonstrated in two metanalyses of observational and randomized controlled trials, respectively [41, 42]. A treatment-induced reduction of albuminuria of >30% is favorable in relation to both cardiovascular and kidney events and such associations have been demonstrated with blockers of the renin-angiotensin system [43–45], SGLT2 inhibitors [46] and GLP-1 receptor agonists [47] among others. This target of albuminuria reduction is now included in the updated ADA guidelines for the treatment of diabetes. That said, there is however a lack of clinical studies using albuminuria as a treatment target, with consecutive addition of treatments with the aim of maximal albuminuria reduction, similar to what is done with blood pressure.

## 4. Implementation

Even though albuminuria testing is a noninvasive, noncomplicated test that adds important knowledge for both risk prediction and for monitoring of treatment effect, there is considerable room for improvement of albuminuria monitoring in CKD. Several global reports indicate a low percentage of albuminuria measurements in populations with type 2 diabetes. A recent survey across 24 primary care organizations in the United States found that a median of 52.9% of the individuals with type diabetes had the recommended annual samples collected. In Denmark, there are signs of improved albuminuria measurements in primary care, as an analysis of repeated cross-sectional studies shows an increase in annual samples from 57.8% in 2012 to 82.7% in 2020 [48]. In nondiabetic CKD however, it seems as if albuminuria testing is much less frequent. In the large CURE-CKD registry in California, comprising more than 2 million individuals with and without diabetes, albuminuria or proteinuria results in individuals with CKD were available in 8.7% and 4.1%, respectively [49]. This calls for an increased focus on albuminuria testing in overall CKD, to improve diagnosis, risk prediction, and treatment selection. In the same publication [49], it was also reported that a renin-angiotensin inhibitor was prescribed to 20.6%, which is far from optimal.

Even with recent updates in guidelines and significant improvements in CVD and CKD protection seen in randomized controlled trials, implementation and uptake of new therapies is slow. One example is the recent CVD protective focus on type 2 diabetes, where SGLT2i and GLP-1RA are recommended when type 2 diabetes and manifest CVD coexists. In a Danish register-based study [50], the uptake of these drugs in this high-risk group was as low as 18%, pointing to a need for the education of healthcare professionals and patients. Similar findings in type 2 diabetes have been demonstrated in the UK general practice database [51], and in global settings [52]. From a kidney protection perspective, it will be interesting to learn whether the current change of guidelines can be implemented quickly across a number of medical specialties and between primary and secondary health care.

## 5. Discussion

Is this disease mongering? Disease mongering is an expression attributed to the medical journalist Lynn Payer describing a broadening of the definition of a treatable disease in order to increase demand for medicinal products and services.

One could argue that health professionals pointing to a large undetected group of people with a silent but chronic condition and at the same time advocating for medical treatment, could just be trying to expand the use and indication for existing drugs. This is not our intention with this paper. Particularly earlier identification of patients with chronic illness could lead to erosion of treatment benefits due to the implementation of treatment in patients with limited risk overall. Raising awareness of the possibility of treatment leading to prevention of ensuing illness is however an important element of public health education and an invaluable means of enhancing professional and public understanding of specific diseases and promoting appropriate uptake of novel therapies. As such, an appropriate balance is indicated.

In that sense, it is important to remember that the CKD classification is not age-adjusted, and widespread screening may lead to the potential overdiagnosing of elderly people with a benign prognosis [53]. This calls for updated guidelines for referral and treatment of elder subjects with impaired kidney function.

We argue that end-stage kidney disease, although developing slowly and with few symptoms, is a grave condition with a severe impact on quality of life, on survival, and is associated with extensive comorbidity and health care costs. Therefore, the implementation of effective medical treatment for people at risk of developing ESKD must be considered, especially if it causes little or no harm. Whether this implementation of treatment should be accompanied by population-wide screening programs, might seem logical and tempting, but it is not clear from the literature that screening efforts lead unequivocally to a better outcome, and clearly more research is needed in this area. Few studies have examined the value of early widespread screening for proteinuria. Boulware et al. analyzed the cost-benefit of annual screening for proteinuria at age 50 years using a Markov model. They concluded that early detection of urine protein followed by treatment with an ACEi or ARB to slow the progression of CKD and decrease mortality is not cost-effective unless selectively directed toward high-risk groups (older persons and persons with hypertension) [54]. As a long-term effect of the current standard treatment of diabetes and CKD, i.e. blocking of the renin-angiotensin system, remains unaddressed, an interesting study has tried to model the possible impact of “early” and “late” intervention [55]. Using data from available randomized clinical trials, it was found that “early” intervention with angiotensin II receptor blockade in a typical subject of 60 years of age, would delay the onset of ESKD by 4.2 years as opposed to 1.4 years, if treatment was initiated at a late stage. This was even more pronounced if patients were younger.

The cost of CKD, and especially ESKD, is high. A large study from Spain [56] documented that in contrast to what many may think, medication costs averaged only 6.6% of the total healthcare cost, with cardiovascular hospitalizations conversely accounting for 77% of total healthcare expenditure in this group. In addition, people on dialysis are associated with higher healthcare costs than people with a kidney transplant. The health care cost of a typical dialysis patient in Denmark is approximately €90,000 annually, provided by the general health care system.

It is clear that CKD is quickly becoming one of the major chronic diseases globally, with a very silent growth that needs attention. Although it can be debated whether screening would be of benefit, it will in any case be necessary for clinicians both in primary and secondary care to try to optimize the selection of CKD patients in whom further work-up and treatment to delay the progression of kidney function decline is appropriate. Particularly, current guidance for treatment selection from the initial workup with biochemistry, urine analysis, and perhaps imaging remains limited in all but a few patients with specific symptoms and findings (i.e., polycystic kidney disease, IgA-nephropathy, and systemic diseases with kidney involvements).

Research is however ongoing with regard to identifying more specific markers for earlier diagnosis of CKD or perhaps even just the risk of developing CKD. New techniques known as “omics” or sometimes referred to as systems medicine are being explored to better understand complex metabolic pathways, often with multiple biomarkers analyzed with proteomics, metabolomics, lipidomics, or genomics [57]. As the biological samples analyzed with these techniques yield a high number of data points, specialized computer software and interpretation are needed, making it unsuitable for everyday clinical practice, but so far mostly for discovering new pathways of disease. One recent example of early diagnosis of diabetic kidney disease is the use of urinary proteomics. A urine sample from an individual with type 2 diabetes with no signs of kidney damage can be analyzed for several thousand peptides and collagen fragments, showing a distinct and previously defined risk pattern for later development of microalbuminuria and CKD [58]. The presence of this “risk pattern” has been demonstrated years before the onset of microalbuminuria. The concept has also been tested prospectively in the PRIORITY study [59], where the urinary proteomic risk pattern could select people at risk for the later development of microalbuminuria. Similar approaches are being tested with other techniques as plasma proteomics [60]and lipidomics [61]. There is still, however, considerable work needed to be done before these techniques become validated and widely available for all types of kidney disease.

Earlier and perhaps more specific diagnoses could contribute to a more differentiated treatment approach. As there is variation in progression rate, there is also a need to select some for more aggressive treatment while others can be monitored solely. This distinction is currently difficult and would probably benefit from well-validated precision techniques as discussed above. Not only is there a need for guidance on which individuals to select for treatment but there is also the topic of what treatment to select. The current approach describes a few general pharmacological classes of drugs with evidence of kidney protection but is used in a very generalized way and often in the late stage of CKD. Much like antihypertensive treatment, it would be great to start therapy early to be able to prevent progression, but also to be able to select therapy in a more modern way, guided by biomarkers or a detailed understanding of the underlying pathology. In late-stage CKD the kidney biopsy is the ultimate diagnostic tool, which sometimes leads to a more targeted therapy, but widespread use is limited by safety concerns. The hope is that the kidney biopsy in the future can be replaced by a “liquid” biopsy, by use of validated use of “omics” or similar techniques and provide early and widespread guidance on CKD pathology leading to more targeted treatment choices. Randomized trials using this approach are however still lacking in CKD.

In addition, the patient with little or no symptoms and therefore undetected or perhaps newly detected CKD is also challenged by the organization of health care. As an example, an individual with type 2 diabetes and diabetic kidney disease may well be involved with different principal caretakers over the many years this condition is present. Being sent back and forth between primary care, specialist diabetes care, and perhaps also the nephrologist will sometimes be complicated and confusing for the patient. A special effort to ensure alignment of patient education and sharing of medical records must be made. Indeed, joint diabetes and nephrology outpatient clinics may prove to benefit the patient, and perhaps even broaden the perspectives of the involved specialists. Joint guidelines are also helping to ensure the best care for the complicated patient with CKD and comorbidity. In the future, primary care physicians can learn from discussing shared cases with specialists, evolving their understanding of CKD, and nephrologists can perhaps develop from being “end-stage” specialists to also contribute to the prevention of CKD and related comorbidity as CVD. Notwithstanding there is much work to do to secure a future with high-quality treatment and prevention of CKD.

## 6. Conclusion

CKD prevalence is increasing but preventive treatment has a great potential. Greater awareness and appropriate screening are necessary first steps to try to avoid a future increase in CKD morbidity and healthcare costs. A large part of this will take place in primary care settings.

## Figures and Tables

**Table 1:** Recommended pharmacological treatments for CKD.

Treatment	General population	Type 2 diabetes	Type 1 diabetes	Heart failure	Potential side effects
ACEi/ARB	X	X	X	X	Hyperkalemia, hypotension, and cough (ACEi)
SGLT2i	X	X		X	Polyuria, genital infections (diabetes), and ketoacidosis (diabetes)
ns-MRA		X			Hyperkalemia

ACEi: angiotensin-converting enzyme inhibitor; ARB: angiotensin II receptor blocker; SGLT2i: sodium–glucose cotransporter 2 inhibitors; ns-MRA: nonsteroidal mineralocorticoid receptor antagonist.

## Data Availability

No data are available for this review.
